# Antibodies Enhance the Suppressive Activity of Extracellular Vesicles in Mouse Delayed-Type Hypersensitivity

**DOI:** 10.3390/ph14080734

**Published:** 2021-07-27

**Authors:** Katarzyna Nazimek, Eugenio Bustos-Morán, Noelia Blas-Rus, Bernadeta Nowak, Justyna Totoń-Żurańska, Michał T. Seweryn, Paweł Wołkow, Olga Woźnicka, Rafał Szatanek, Maciej Siedlar, Philip W. Askenase, Francisco Sánchez-Madrid, Krzysztof Bryniarski

**Affiliations:** 1 Department of Immunology, Jagiellonian University Medical College, 18 Czysta St., 31-121 Krakow, Poland; katarzyna.nazimek@uj.edu.pl (K.N.); bernadeta.nowak@uj.edu.pl (B.N.); 2 Department of Immunology, Hospital de la Princesa, Health Research Institute of Princesa Hospital (ISS-IP), Autonomous University of Madrid, 28006 Madrid, Spain; ebustosmoran@gmail.com (E.B.-M.); noe.blas2@gmail.com (N.B.-R.); fsmadrid@salud.madrid.org (F.S.-M.); 3 Section of Rheumatology, Allergy and Clinical Immunology, Yale University School of Medicine, New Haven, CT 208011, USA; philip.askenase@yale.edu; 4 Center for Medical Genomics OMICRON, Jagiellonian University Medical College, 31-034 Krakow, Poland; justyna.toton-zuranska@uj.edu.pl (J.T.-Ż.); michal.seweryn@wmii.uni.lodz.pl (M.T.S.); pawel.wolkow@uj.edu.pl (P.W.); 5 Department of Cell Biology and Imaging, Institute of Zoology and Biomedical Research, Jagiellonian University, 30-387 Krakow, Poland; olga.woznicka@uj.edu.pl; 6 Department of Clinical Immunology, Institute of Paediatrics, Jagiellonian University Medical College, 30-663 Krakow, Poland; rafal.szatanek@uj.edu.pl (R.S.); misiedla@cyf-kr.edu.pl (M.S.)

**Keywords:** antigen-presenting cells, antigen-specific T cell suppression, contact hypersensitivity, delayed-type hypersensitivity, extracellular vesicles, immune tolerance, intercellular communication, macrophages, miRNA-150, therapeutic activity of exosomes

## Abstract

Previously, we showed that mouse delayed-type hypersensitivity (DTH) can be antigen-specifically downregulated by suppressor T cell-derived miRNA-150 carried by extracellular vesicles (EVs) that target antigen-presenting macrophages. However, the exact mechanism of the suppressive action of miRNA-150-targeted macrophages on effector T cells remained unclear, and our current studies aimed to investigate it. By employing the DTH mouse model, we showed that effector T cells were inhibited by macrophage-released EVs in a miRNA-150-dependent manner. This effect was enhanced by the pre-incubation of EVs with antigen-specific antibodies. Their specific binding to MHC class II-expressing EVs was proved in flow cytometry and ELISA-based experiments. Furthermore, by the use of nanoparticle tracking analysis and transmission electron microscopy, we found that the incubation of macrophage-released EVs with antigen-specific antibodies resulted in EVs’ aggregation, which significantly enhanced their suppressive activity in vivo. Nowadays, it is increasingly evident that EVs play an exceptional role in intercellular communication and selective cargo transfer, and thus are considered promising candidates for therapeutic usage. However, EVs appear to be less effective than their parental cells. In this context, our current studies provide evidence that antigen-specific antibodies can be easily used for increasing EVs’ biological activity, which has great therapeutic potential.

## 1. Introduction

Recent advances in studies on the biology of extracellular vesicles (EVs) demonstrated their exceptional role in intercellular communication [1], both in physiological and pathological conditions [2]. Among other processes, EV-mediated cell signaling cascades are currently extensively investigated in the terms of immune regulation. EVs have also been proposed to substitute for the activity of parental immune cells; however, they seem to be less effective [3]. At present, EVs receive special attention as physiological delivery tools, the usage of which reduces the side effects of treatment. However, the latter application is still fraught with many challenges, including enhancing their biological effectiveness and directing them towards desired target cells [4].

Shortly after the discovery of suppressor T (Ts) cells, one of their subpopulations was shown to inhibit mouse hapten-induced contact hypersensitivity (CHS) reaction by generating so-called T suppressor factor (TsF) [5,6]. Our recent research uncovered that TsF consists of miRNA-150 carried by EVs, hereinafter called Ts-EVs. Those downregulate both hapten-induced CHS [5,7,8], and delayed-type hypersensitivity (DTH) to protein antigens, such as ovalbumin (OVA) [9], and casein [10]. Both miRNA-150 and Ts-EVs are produced by CD8+ Ts cells, not expressing FoxP3, and activated through the intravenous administration of syngeneic red blood cells coupled with hapten or protein antigen [5]. Interestingly, Ts-EVs are surface coated with antigen-specific antibody light chains derived by B1a cells activated by skin immunization [7,11]. This ensures the antigen specificity of immune suppression mediated by Ts-EV-delivered miRNA-150 [12].

Our subsequent detailed studies revealed that miRNA-150-carrying Ts-EVs target antigen-presenting cells (APCs), especially antigen-primed macrophages, both in hapten-induced CHS and in OVA-induced DTH reactions [8,9]. In turn, Ts-EV-targeted macrophages suppress DTH immune responses by inhibiting the activation and proliferation of effector T lymphocytes and by increasing their apoptosis [8,13]. In addition, macrophages treated with TsF were previously shown to release the macrophage suppressor factor (MSF) of barely characterized nature [6]. Moreover, Tung et al. have recently demonstrated that regulatory T cell-derived EVs induce tolerogenic phenotype in targeted dendritic cells due to the transmission of miRNA-150 [14]. Together with our observations, this implies a crucial role of miRNA-150 in tolerogenic interactions between regulatory/suppressor T lymphocytes and APCs. However, this speculation remained unclear, and thus the current studies aimed at investigating the exact mechanism of suppressive action of Ts-EV-targeted macrophages on effector T cells.

To examine how APCs treated with Ts-EVs suppress effector T lymphocytes, we cultured Ts-EV-pretreated macrophages and tested the resulting supernatant for suppressive activity in vivo, showing that the DTH suppression is mediated by macrophage-derived EVs, hereinafter called Mac-EVs. Furthermore, the suppressive action of Mac-EVs was found to be miRNA-150-dependent, triggered by immune synapse formation, and could be either abolished by pre-incubation with anti-CD9 antibodies or enhanced by pre-incubation with antigen-specific antibodies that can specifically bind to Mac-EVs. The latter finding led us to hypothesize that antigen-specific antibodies aggregate Mac-EVs expressing major histocompatibility complex (MHC) class II molecules. The final validation of this assumption with nanoparticle tracking analysis (NTA), transmission electron microscopy (TEM), and in vivo assays, confirmed the significantly enhanced suppressive activity of aggregated Mac-EVs against DTH effector T cells. To the best of our knowledge, this is the first demonstration that antigen-specific antibodies could be easily used for increasing the biological activity of MHC class II-positive EVs, which appears to have a great therapeutic potential, both in enhancing EVs’ effectiveness and directed cell targeting.

## 2. Results

### 2.1. CHS Effector Cells Are Suppressed by CD9^pos^ EVs Enriched from the Culture of Ts-EV-Treated Macrophages

**Figure 1:**
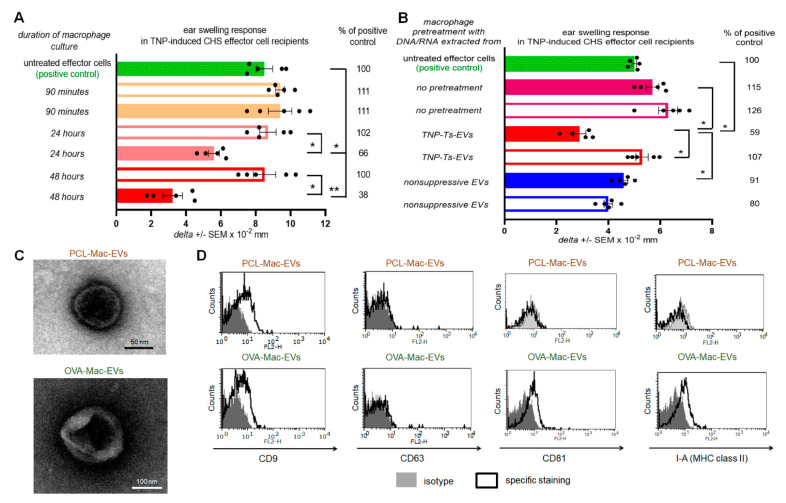
Macrophages treated with Ts-EVs release inhibitory Mac-EVs that differ in marker’s expression pattern depending on mouse immunizing antigen. (**A**) Macrophages, after 30 min treatment at 37 °C with TNP-Ts-EVs, were cultured in protein-free MDM medium. Yielded supernatant was collected 90 min, 24 h or 48 h later, filtered and ultracentrifuged, and resulting fractions (i.e., pellet–filled bars; and supernatant above–open bars) were used to treat CHS effector cells prior to their transfer into naive recipients (*n* = 5 per group) that were immediately challenged with hapten to elicit CHS reaction, measured as ear swelling 24 h later. (**B**) Untreated macrophages or macrophages treated for 30 min at 37 °C with DNA/RNA extracted from either TNP-Ts-EVs or control, non-suppressive EVs, were cultured in protein-free MDM medium for 48 h. Yielded supernatant was filtered and ultracentrifuged, and resulting fractions (i.e., pellet–filled bars; and supernatant above–open bars) were used to treat CHS effector cells prior to their transfer into naive recipients (*n* = 5 per group) that were immediately challenged with hapten to elicit CHS reaction, measured as ear swelling 24 h later. (**C**) PCL-Mac-EVs (produced by TNP-Ts-EV-treated macrophages from PCL-sensitized mice) and OVA-Mac-EVs (produced by OVA-Ts-EV-treated macrophages from OVA-immunized mice) were absorbed onto cupper grid, negatively stained with 3% uranyl acetate, and visualized with TEM microscope. (**D**) PCL-Mac-EVs and OVA-Mac-EVs were coated onto latex beads, stained with fluoresceinated antibodies against selected EVs’ markers, including CD9, CD63, CD81 tetraspanins and I-A molecules, and analyzed with flow cytometry. Data are expressed as *delta* ± SEM. One-way ANOVA with post hoc RIR Tukey test; * *p* < 0.05, ** *p* < 0.01.

From our previous experiments we knew that the successful induction of Ts cell-mediated suppression of CHS and DTH reactions in mice requires both tolerization with hapten- or protein antigen-conjugated syngeneic red blood cells and sensitization with the same hapten or protein antigen [5,9]. Thus, we cultured Ts-EV-treated macrophages isolated from picryl chloride (PCL)-sensitized mice in protein-free medium and collected the resulting supernatants after 90 min, 24 h and 48 h of the culture. Then, culture supernatants were ultracentrifuged and both the pellet and supernatant above were recovered and tested for potential suppressive activity in vivo, in an adoptive transfer of PCL-induced CHS effector cells. The suppression of the subsequently elicited CHS ear swelling response was observed in recipients of CHS effector cells pre-incubated with the pellet from ultracentrifuged supernatant collected from 24- and 48-h culture of Ts-EV-treated macrophages, while other supernatant preparations failed to modulate CHS reaction (Figure 1A). Then, we cultured macrophages from PCL-sensitized mice that had been treated with DNA/RNA extracted from either trinitrophenol (TNP)-Ts-EVs or control non-suppressive EVs [5], or that had been remained untreated, and collected the resulting supernatants after 48 h. After ultracentrifugation, both the pellets and supernatants above were used to treat CHS effector cells prior to their adoptive transfer. Significant suppression of the CHS ear swelling response was only observed in recipients of the effector cells treated with ultracentrifugation pellet from the culture of macrophages pre-incubated with DNA/RNA from TNP-Ts-EVs (Figure 1B). Thus, we concluded that Ts-EV-treated macrophages release the suppressive factor that could be pelleted from the culture supernatant by ultracentrifugation. The latter observation suggested that CHS suppression could be mediated by macrophage-derived EVs. To verify this speculation and to assess whether it is also true in the suppression of protein antigen-induced DTH reaction, we cultured macrophages from PCL-sensitized or OVA-immunized CBA mice that had been pretreated with TNP-Ts-EVs or OVA-Ts-EVs, respectively, and ultracentrifuged the resulting supernatants collected after 48 h. The yielded pellets were then visualized with TEM and, after coupling onto latex beads and staining with fluoresceinated antibodies, analyzed cytometrically. TEM microscopic analysis revealed the presence of small EVs in pellets from the culture supernatant produced by both TNP-Ts-EV-treated and OVA-Ts-EV-treated macrophages, which we decided to call PCL-Mac-EVs and OVA-Mac-EVs, respectively (Figure 1C). Interestingly, we found the difference in the expression of EV markers between PCL-Mac-EVs and OVA-Mac-EVs. Namely, PCL-Mac-EVs expressed only CD9 tetraspanin, while OVA-Mac-EVs displayed CD9 and CD81 tetraspanins along with I-A (MHC class II) molecules (Figure 1D).

### 2.2. Generation and Suppressive Activity of Mac-EVs Both Depend on miRNA-150

**Figure 2:**
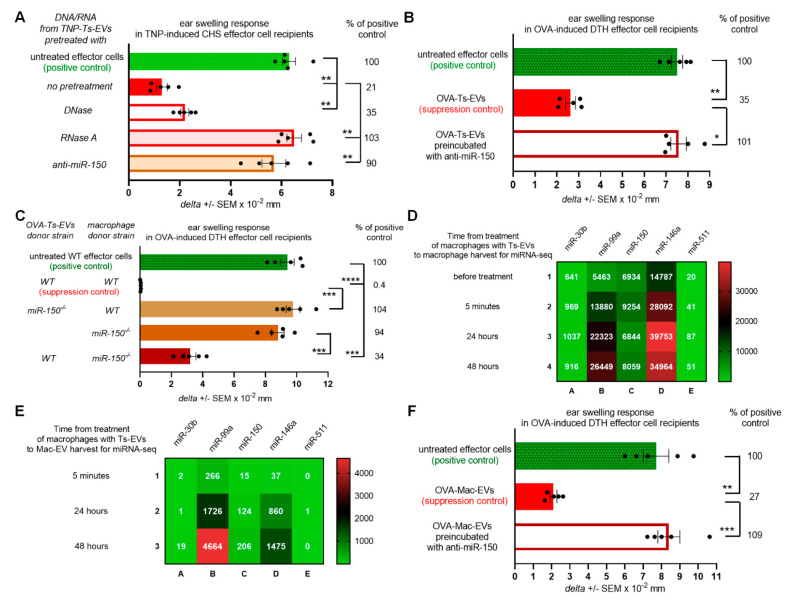
Ts-EV-treated macrophages release Mac-EVs inhibiting DTH in miRNA-150-dependent manner. (**A**) CHS effector T cells and macrophages were incubated with TNP-Ts-EV-extracted DNA/RNA pretreated with DNase, RNase A or anti-miR-150, and then adoptively transferred to naive recipients (*n* = 5 per group) that were immediately challenged with hapten to elicit CHS reaction, measured as ear swelling 24 h later. (**B**) DTH effector T cells and macrophages were incubated with OVA-Ts-EVs, where indicated pretreated with anti-miR-150, and then adoptively transferred to naive recipients (*n* = 5 per group) that 24 h later were challenged with OVA to elicit DTH reaction, measured as ear swelling 24 h later. (**C**) DTH effector cells were treated with either OVA-Mac-EVs from wild type mice, EVs from macrophages treated with OVA-Ts-EVs from miRNA-150^−/−^ mice, EVs from untreated miRNA-150^−/−^ mouse macrophages, or with EVs from miRNA-150^−/−^ mouse macrophages treated with OVA-Ts-EVs from wild type mice. Twenty four hours later recipients of adoptively transferred DTH effector cells (*n* = 5 per group) were challenged with OVA to elicit DTH reaction, measured as ear swelling 24 h later. (**D**) RNA extracts from untreated macrophages or macrophages treated with OVA-Ts-EVs were subjected to miRNA deep sequencing (*n* = 1 per time-point). (**E**) RNA extracts from OVA-Mac-EVs collected from 5 min-, 24 h- or 48 h- macrophage culture supernatant were subjected to miRNA deep sequencing (*n* = 1 per time-point). (**F**) DTH effector cells were incubated with OVA-Mac-EVs, where indicated pretreated with anti-miR-150, and then adoptively transferred to naive recipients (*n* = 5 per group) that 24 h later were challenged with OVA to elicit DTH reaction, measured as ear swelling 24 h later. Data are expressed as *delta* ± SEM. One-way ANOVA with post hoc RIR Tukey test; * *p* < 0.05, ** *p* < 0.01, *** *p* < 0.005, **** *p* < 0.001.

From the previous studies we knew that macrophages present among CHS effector cells are targeted by Ts-EVs [8]. Therefore, we assumed that their suppressive activity is induced by Ts-EV-enclosed miRNA-150, which had been formerly found to mediate the regulatory activity of Ts-EVs [5]. To initially verify this hypothesis, we incubated CHS effector cell mixture containing both macrophages and T cells with DNA/RNA extracted from Ts-EVs that had been pretreated with either DNase, RNase A or single strand antisense oligonucleotides for miRNA-150 (i.e., anti-miR-150), prior to their adoptive transfer. Subsequently, CHS ear swelling response was significantly suppressed in recipients of CHS effector cells pretreated with either intact or DNase-pretreated DNA/RNA extract of Ts-EVs, while treatment with RNase A and anti-miR-150 abolished the suppressive activity of Ts-EV-extracted DNA/RNA (Figure 2A). Analogous results were observed in recipients of OVA-induced DTH effector T cells and macrophages incubated with OVA-Ts-EVs pretreated with anti-miR-150 (Figure 2B). Both findings confirmed the assumed crucial role of miRNA-150 in the induction of macrophage suppressive function mediated by Mac-EVs. To ultimately confirm this hypothesis, we incubated DTH effector cells with OVA-Mac-EVs released by macrophages that had been isolated from OVA-immunized miRNA-150^−/−^ mice and pretreated with OVA-Ts-EVs from wild type mice. Indeed, these OVA-Mac-EVs also suppressed DTH reaction. In contrast, EVs released by untreated miRNA-150^−/−^ mouse macrophages and, especially, EVs from wild type mouse macrophages pretreated with OVA-Ts-EVs from miRNA-150^−/−^ mice were non-suppressive (Figure 2C). These results confirmed that Ts-EV-enclosed miRNA-150 induces macrophages to release the suppressive Mac-EVs.

However, our attention was drawn to the observation that OVA-Mac-EVs derived by miRNA-150^−/−^ mouse macrophages were less inhibitory than those from wild type mice (Figure 2C). Thus, we speculated that macrophage-derived miRNA-150 may be involved in suppressive activity of Mac-EVs, in addition to Ts-EV-transmitted miRNA-150. In the pilot study performed to examine this hypothesis, we cultured macrophages from OVA-immunized wild type mice that were pretreated with OVA-Ts-EVs and subjected the cells and their derived OVA-Mac-EVs harvested at different time-points to miRNA deep sequencing. By means of the Principal Component Analysis (PCA) we showed that the macrophage and EV samples may be well discriminated using the miRNA expression profiles, by the first principal component (PC1, Figure S1). This allowed the downstream analysis of miRNAs expressed both in macrophages and Mac-EVs. Among all detected sequences, we focused on miRNA-150 as well as on miRNAs involved in macrophage differentiation and polarization that had been shown to inhibit the downstream functionality of these cells [15,16]. Four miRNAs (i.e., miRNA-30b, miRNA-99a, miRNA-146a and miRNA-511) involved in the differentiation of M2 macrophages were expressed in Ts-EV-pretreated macrophages (Figure 2D). In addition, three of them, namely miRNA-30b, miRNA-99a and miRNA-146a, were also expressed in Mac-EVs (Figure 2E). When analyzing the miRNA-150 expression pattern, we found an increased number of miRNA-150 copies in macrophages collected 5 min after treatment with Ts-EVs, when compared to untreated macrophages (Figure 2D). Then, the number of miRNA-150 copies decreased for macrophages collected after 24 h (to a level comparable to untreated macrophages) to increase again in macrophages harvested after 48 h (Figure 2D). On the other hand, the number of miRNA-150 copies in OVA-Mac-EVs was higher at each subsequent time-point, reaching the highest number for OVA-Mac-EVs harvested after 48 h of culture (Figure 2E), when they express the most efficient inhibitory activity (Figure 1A). This prompted us to examine the possible involvement of miRNA-150 in Mac-EV-mediated suppression by incubating OT-II mouse OVA-Mac-EVs with anti-miR-150 prior to treating OVA-specific DTH effector cells from OT-II mice that have been then adoptively transferred to wild type recipients. The incubation of OVA-Mac-EVs with anti-miR-150 completely abolished their inhibitory activity (Figure 2F), providing evidence for the key role of miRNA-150 in Mac-EV-mediated suppression of DTH reaction. It is worth noting that attempts to inhibit miRNA-99a in OVA-Mac-EVs, which was the most abundant among the analyzed sequences at 48 h (Figure 2E), failed to alter the suppressive activity of Mac-EVs (Figure S2). Thus, these overall observations proved the principal role of miRNA-150 in both the induction of Mac-EV-release by Ts-EV-pretreated macrophages and the downstream inhibitory activity of Mac-EVs.

### 2.3. At the Immune Synapse, Ts-EVs Modulate Vesicle-Mediated Interaction of Raji B Cells and Jurkat T Cells

Recent studies on EV-mediated intercellular signaling during immune synapse formation revealed the unidirectional transfer of CD63^pos^ EVs from intact Jurkat T cells to SEE-pulsed Raji B cells acting as APCs [17]. The expression of MHC class II by OVA-Mac-EVs (Figure 1D) led us to hypothesize that they might also be transferred by Ts-EV-targeted APCs to effector T cells at the immune synapse.

miRNA sequences are conservative among species. Accordingly, the sequence of mouse Ts-EV-enclosed miRNA-150 [5], precisely mmu-miRNA-150-5p, that induces macrophages to release Mac-EVs (Figure 2), has exactly the same nucleotide order and composition as human hsa-miRNA-150-5p, according to miRBase (http://www.mirbase.org/, accessed date: 29 March 2021). This allowed subjecting OVA-Ts-EVs to the abovementioned standardized model of immune synapse formation by Raji and Jurkat cells, assessed by cytometric analysis and confocal microscopy.

**Figure 3:**
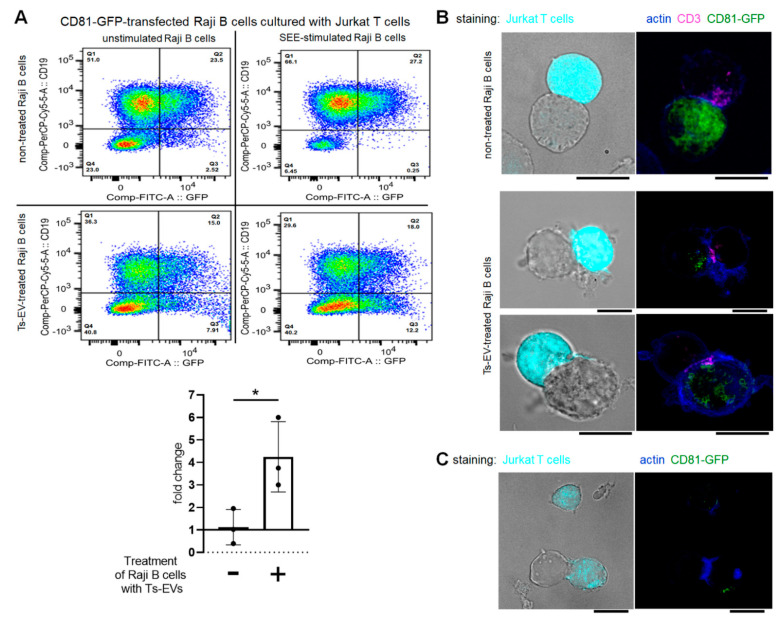
OVA-Ts-EVs modulate vesicle-mediated interaction of Raji B cells and Jurkat T cells at the immune synapse. (**A**) CD81-GFP-transfected Raji B cells were pulsed with SEE superantigen for 30 min at 37 °C and then cultured with Jurkat T cells in the presence of OVA-Ts-EVs. Twenty four hours later, cells were stained with viability dye and fluoresceinated antibodies against CD19, and analyzed with flow cytometry (*n* = 3). Relative changes in the percentage of CD19^neg^GFP^pos^ Jurkat T cells caused by SEE-stimulation of Raji B cells were calculated as follows: (percentage of CD19^neg^GFP^pos^ events in SEE-stimulated sample)/(percentage of CD19^neg^GFP^pos^ events in unstimulated sample); and shown in the graph. (**B**) CD81-GFP-transfected Raji B cells were left untreated (upper panel) or were treated with OVA-Ts-EVs for 4 h at 37 °C (lower panel), both populations were then pulsed with SEE for 30 min at 37 °C, and mixed with CMAC-stained Jurkat T cells (1 × 10^5^ cells) in a ratio 1:1. Then, cell mixtures were plated onto Poly-L-Lys-coated slides for 1 h incubation at 37 °C, fixed, blocked, stained with selected primary and then secondary antibodies, mounted on Prolong Gold and analyzed with confocal microscope; scale bar: 10 µm. (**C**) CD81-GFP-transfected Raji B cells were treated with OVA-Ts-EVs for 4 h at 37 °C, pulsed with SEE for 30 min at 37 °C, and cultured with CMAC-stained Jurkat T cells (1 × 10^5^ cells) in a ratio 1:1 for 24 h on standard culture plate. Then, cell mixtures were plated onto Poly-L-Lys-coated slides for 1 h incubation at 37 °C, fixed, blocked, stained with selected primary and then secondary antibodies, mounted on Prolong Gold and analyzed with confocal microscope; scale bar: 10 µm. Data are expressed as mean ± SD. Two-tailed Student *t*-test; * *p* < 0.05.

A pilot study showed that OVA-Ts-EVs failed to impact the transfer of vesicles between CD63-GFP-transfected Raji B cells and Jurkat T cells (data not shown). Since Mac-EVs have been shown to express CD81 but not CD63 (Figure 1D), we decided to employ the CD81-GFP-transfected Raji B cells for further study. We observed a significant increase in the percentage of CD19^neg^GFP^pos^ cells caused by SEE-stimulation when immune synapses were formed by CD81-GFP-transfected, Ts-EV-treated Raji B cells and Jurkat T cells. In contrast, SEE-stimulation failed to change the percentage of CD19^neg^GFP^pos^ cells when immune synapses were formed by cells untreated with Ts-EVs (Figure 3A). Additionally, OVA-Ts-EVs have no impact on EV-mediated interactions between CD81-GFP-transfected Jurkat T cells and Raji B cells (data not shown). Thus, we assumed that CD81-GFP-transfected, Ts-EV-treated Raji B cells can transfer CD81^pos^ vesicles to Jurkat T cells at the immune synapse. To further validate this assumption, we cultured CD81-GFP-transfected, SEE-pulsed Raji B cells, in some instances after treatment with Ts-EVs, with Jurkat T cells either for an hour or for 24 h, and then visualized the cells with a fluorescence confocal microscope. When an immune synapse was formed by SEE-pulsed Raji B cells that had not been treated with Ts-EVs, the CD81-GFP-emitted green fluorescence signal seems to be equally dispersed within the whole cell (Figure 3B, upper panel). On the contrary, the CD81-GFP-emitted green fluorescence in Ts-EV-treated Raji B cells appears to be condensed close to the immune synapse area detected by CD3 accumulation in Jurkat T cells, marked in magenta (Figure 3B, lower panel). Moreover, after 24 h of co-culture of CD81-GFP-transfected, Ts-EV-treated, SEE-pulsed Raji B cells with Jurkat T cells, we found that some of the latter cells expressed GFP-emitted fluorescence (Figure 3C). Altogether, these observations greatly support the conclusion that the Ts-EV-induced release of APC-derived EVs is the most effective at the immune synapse.

### 2.4. Suppressive Activity of PCL-Mac-EVs Is Abolished by Incubation with Anti-CD9 Antibodies but Not with Anti-TNP Antibodies

**Figure 4:**
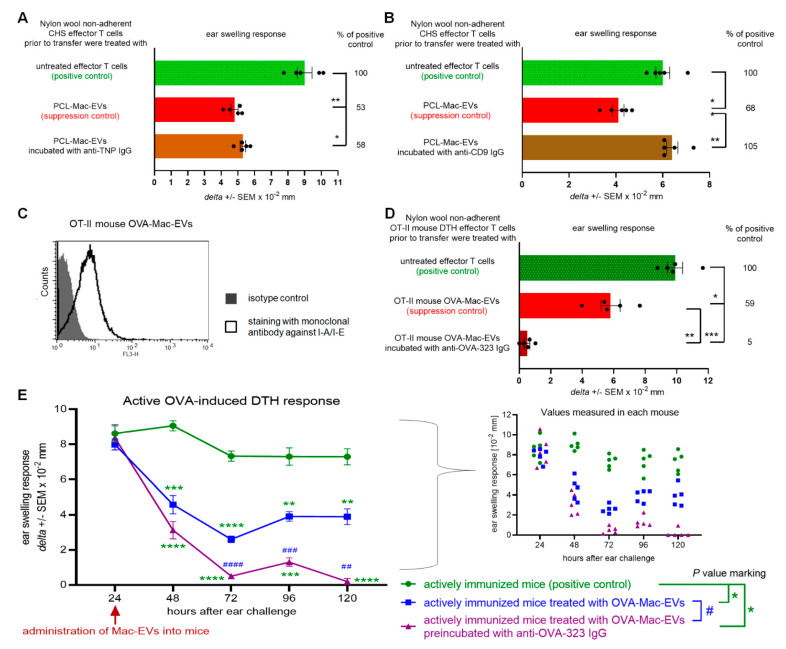
Antibodies modulate the suppressive activity of PCL- and OVA-Mac-EVs. (**A**) CHS effector T cells were incubated for 30 min at 37 °C with PCL-Mac-EVs, where indicated pre-incubated with anti-TNP IgG antibodies. Afterwards, CHS effector T cells were adoptively transferred to naive recipients (*n* = 5 per group) that were immediately challenged with hapten to elicit CHS reaction, measured as ear swelling 24 h later. (**B**) CHS effector T cells were incubated for 30 min at 37 °C with PCL-Mac-EVs, where indicated pre-incubated with anti-CD9 IgG antibodies. Afterwards, CHS effector T cells were adoptively transferred to naive recipients (*n* = 5 per group) that were immediately challenged with hapten to elicit CHS reaction, measured as ear swelling 24 h later. (**C**) OT-II mouse OVA-Mac-EVs were coated onto latex beads, stained with fluoresceinated antibodies against I-A/I-E molecules, and analyzed with flow cytometry. (**D**) DTH effector T cells from OVA-immunized OT-II mice were incubated for 30 min at 37 °C with OT-II mouse OVA-Mac-EVs, where indicated pre-incubated with anti-OVA-323 IgG antibodies, and then were adoptively transferred to naive wild type recipients (*n* = 5 per group) that 24 h later were challenged with OVA to elicit DTH reaction, measured as ear swelling 24 h later. (**E**) After measuring of 24-h DTH ear swelling, actively immunized C57BL/6 mice (*n* = 5 per group) were administered intraperitoneally with OT-II mouse OVA-Mac-EVs alone or preincubated with anti-OVA-323 IgG antibodies, and subsequent DTH ear swelling was measured up to 120 h after challenge. Data are expressed as *delta* ± SEM. One-way or two-way ANOVA with post hoc RIR Tukey test; * *p* < 0.05, ** *p* < 0.01, *** *p* < 0.005, **** *p* < 0.001, ^#^ *p* < 0.05, ^##^ *p* < 0.01, ^###^ *p* < 0.005, ^####^ *p* < 0.001.

Postulated effective transfer of Mac-EVs at the immune synapse led us to speculate that their T-cell targeting capability is highly specific, and thus may depend on interaction with a particular antigen displayed on Mac-EV surface. Firstly, we attempted to evaluate this speculation by incubating PCL-Mac-EVs from CBA mice with IgG antibodies specific for TNP hapten, which had been used to sensitize macrophage donors. However, this failed to influence the suppressive action of PCL-Mac-EVs on CHS effector T cells (Figure 4A). Consequently, we assumed that T-cell targeting by PCL-Mac-EVs does not depend on hapten-binding, possibly due to the fact that PCL-Mac-EVs do not express MHC class II molecules (Figure 1D), and thus they may not display hapten molecules as well. Therefore, at the next step, we incubated PCL-Mac-EVs with anti-CD9 IgG antibodies, which abolished their inhibitory activity against adoptively transferred CHS effector T cells (Figure 4B). This observation implies a possible role of CD9 tetraspanin in the binding of Mac-EVs to T cells [18].

### 2.5. Suppressive Activity of MHC Class II-Expressing OVA-Mac-EVs Is Enhanced by Incubation with Antigen-Specific Antibodies

To further investigate the specific interactions of Mac-EVs with targeted T cells, we employed OVA-Mac-EVs from OT-II mouse macrophages treated with OVA-Ts-EVs from wild type C57BL/6 mice. Cytometric analysis confirmed the surface expression of MHC class II molecules on OT-II mouse OVA-Mac-EVs (Figure 4C). Then, we incubated them with anti-OVA-323 IgG antibodies and, after washing by ultracentrifugation, used them to treat DTH effector T cells prior to their adoptive transfer. Remarkably, incubation of OT-II mouse OVA-Mac-EVs with anti-OVA-323 IgG antibodies significantly enhanced their inhibitory activity against DTH effector T cells, when compared to intact OT-II mouse OVA-Mac-EVs (Figure 4D). Furthermore, we intraperitoneally injected OT-II mouse OVA-Mac-EVs, ither alone or preincubated with anti-OVA-323 IgG antibodies, into actively immunized mice at the 24-h peak of DTH response, which resulted in a significant reduction in ear swelling, observed up to 120 h after challenge with OVA. Again, pretreatment of OVA-Mac-EVs with anti-OVA-323 antibodies increased their therapeutic activity in DTH symptomatic animals, when compared to intact OVA-Mac-EVs (Figure 4E). These surprising observations prompted us to uncover the mechanism underlying the enhancing effect of anti-OVA-323 IgG antibodies on the suppressive activity of OT-II mouse OVA-Mac-EVs.

### 2.6. Anti-OVA-323 Antibodies Bind to MHC Class II-Expressing OVA-Mac-EVs from OT-II Mice

**Figure 5:**
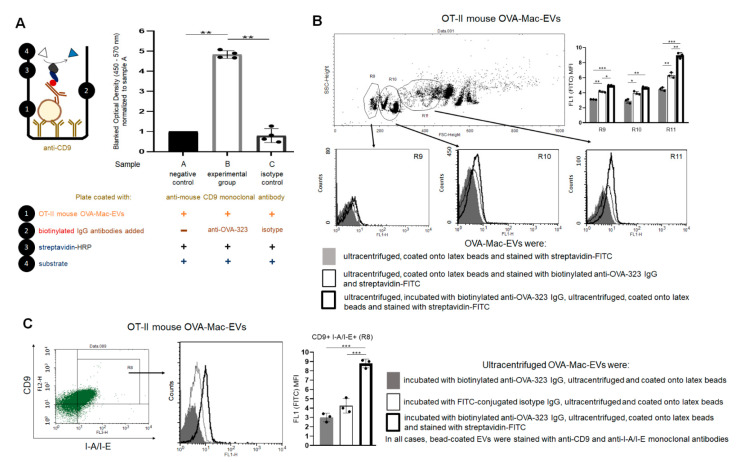
Anti-OVA-323 antibodies specifically bind to OVA-Mac-EVs from OT-II mice. (**A**) OT-II mouse OVA-Mac-EVs were incubated on ELISA plate coated with anti-CD9 antibodies for 2 h at room temperature. Then, biotinylated anti-OVA-323 IgG or biotinylated isotype IgG were added to selected wells, and the plate was incubated overnight at 4 °C. Afterwards, streptavidin-HRP was added to each well, which was followed by 45 min incubation at room temperature and addition of TMB substrate. Reaction was stopped after 5 min with 1 M H_3_PO_4_, and absorbance was measured at 450 nm with a reference wavelength of 570 nm. Then, blanked absorbance values were normalized to mean blanked absorbance detected in wells with added OVA-Mac-EVs but not antibodies (*n* = 4). (**B**) OT-II mouse OVA-Mac-EVs were either incubated with biotinylated anti-OVA-323 IgG overnight at 4 °C, ultracentrifuged, and coated onto latex beads, or coated onto latex beads and incubated with biotinylated anti-OVA-323 IgG for an hour at room temperature. Then, both OVA-Mac-EV preparations were incubated with streptavidin-FITC, and analyzed with flow cytometry (*n* = 3). (**C**) OT-II mouse OVA-Mac-EVs were incubated with either biotinylated anti-OVA-323 IgG, or FITC-conjugated isotype IgG overnight at 4 °C. After ultracentrifugation, both OVA-Mac-EV preparations were coated onto latex beads, incubated with streptavidin-FITC and fluoresceinated antibodies against CD9 and I-A/I-E, and analyzed with flow cytometry (*n* = 3). Data are expressed as mean ± SD. One-way or two-way ANOVA with post hoc RIR Tukey test; * *p* < 0.05, ** *p* < 0.01, *** *p* < 0.005.

First, we established an ELISA-based assay to investigate whether anti-OVA-323 IgG antibodies can actually bind to OVA-Mac-EVs derived by OT-II mouse macrophages. For this purpose, we incubated OVA-Mac-EVs on the ELISA plate coated with anti-CD9 antibodies. After the removal of unbound EVs, we added either anti-OVA-323 or isotype biotinylated IgG antibodies and, after incubation and washing, we detected the IgG antibodies that had bound to OVA-Mac-EVs with streptavidin-HRP. The amount of detected anti-OVA-323 IgG antibodies that bound to OVA-Mac-EVs was significantly higher than that of isotype IgG antibodies (Figure 5A). Thus, this assay revealed that anti-OVA-323 IgG antibodies can strongly bind to OVA-Mac-EVs in contrast to isotype IgGs.

To further confirm the binding capability of anti-OVA-323 antibodies to OVA-Mac-EVs, we either incubated OVA-Mac-EVs overnight with biotinylated anti-OVA-323 IgGs and then coated them onto latex beads, or firstly coated OVA-Mac-EVs onto latex beads and then incubated them with biotinylated anti-OVA-323 IgGs for an hour. Cytometric detection of biotinylated antibodies with streptavidin-FITC showed a stronger fluorescence signal in the case of OVA-Mac-EVs that had been incubated with anti-OVA-323 antibodies prior to coating onto latex beads, and, additionally, the mean fluorescence intensity (MFI) value was found to increase together with FSC value (Figure 5B). To ultimately confirm the specificity of the binding, we incubated the OT-II mouse OVA-Mac-EVs overnight with either biotinylated anti-OVA-323 IgG antibodies or FITC-coupled isotype IgG antibodies, ultracentrifuged them, coated them onto latex beads and stained with fluoresceinated antibodies against CD9 and MHC class II molecules, and with streptavidin-FITC to detect biotinylated anti-OVA-323 IgGs. Further cytometric analysis revealed that anti-OVA-323 antibodies bind to CD9^pos^I-A/I-E^pos^ OVA-Mac-EVs much more strongly than isotype IgGs (Figure 5C). These overall findings allowed us to conclude that binding of antigen-specific antibodies to MHC class II-expressing Mac-EVs significantly enhances the suppressive activity of the latter. However, the mechanism underlying the antibody-induced enhancing effect remained unclear.

### 2.7. Treatment with Anti-OVA-323 IgG Antibodies Causes the Aggregation of OT-II Mouse OVA-Mac-EVs to Enhance Their Suppressive Activity

**Figure 6:**
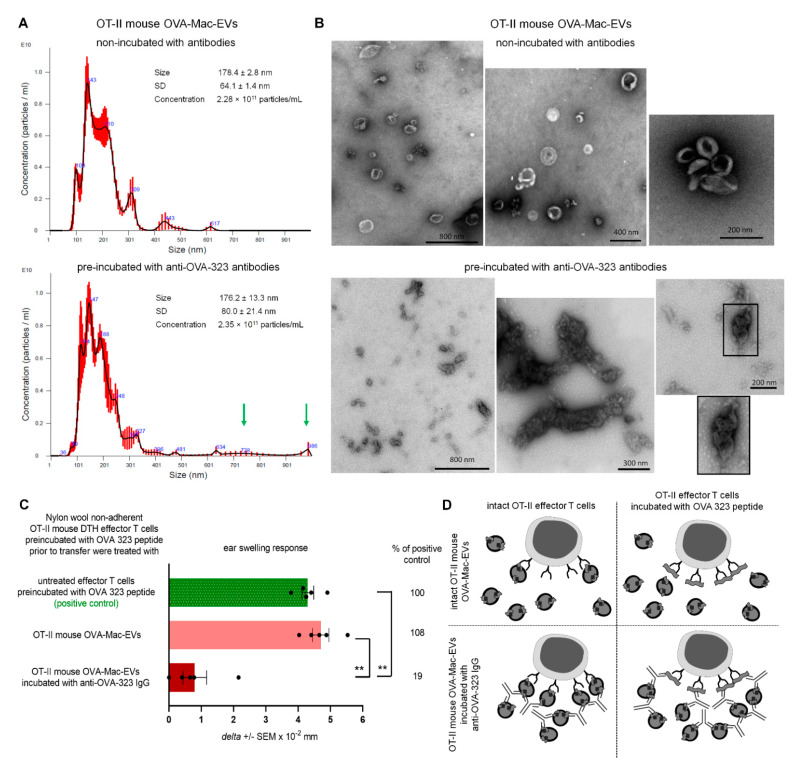
Incubation of OT-II mouse OVA-Mac-EVs with anti-OVA-323 antibodies leads to their aggregation and enhances their suppressive activity. (**A**) OT-II mouse OVA-Mac-EVs were incubated alone or with biotinylated anti-OVA-323 IgG overnight at 4 °C. After ultracentrifugation, both OVA-Mac-EV preparations were subjected to nanoparticle tracking analysis (NTA). (**B**) OT-II mouse OVA-Mac-EVs were incubated alone or with biotinylated anti-OVA-323 IgG overnight at 4 °C. After ultracentrifugation, both OVA-Mac-EV preparations were absorbed onto cupper grid, negatively stained with 3% uranyl acetate, and visualized with TEM microscope. (**C**) OT-II mouse OVA-Mac-EVs were incubated alone or with biotinylated anti-OVA-323 IgG overnight at 4 °C. After ultracentrifugation, both OVA-Mac-EV preparations were used for 30 min treatment at 37 °C of OT-II mouse DTH effector T cells that had been pre-incubated with OVA-323 peptide for 20 min at 37 °C. Then, DTH effector T cells were adoptively transferred to naive wild type recipients (*n* = 5 per group) that 24 h later were challenged with OVA to elicit DTH reaction, measured as ear swelling 24 h later. (**D**) Scheme showing the proposed mechanism of enhancement of OVA-Mac-EVs’ suppressive activity against OVA-specific DTH effector T cells, in some instances pre-incubated with OVA-323 peptide, induced by anti-OVA-323 IgG antibodies. NTA results are shown as mean ± SD, and DTH ear swellings are expressed as *delta* ± SEM. One-way ANOVA with post hoc RIR Tukey test; ** *p* < 0.01.

When assessing the binding of OVA-323-specific antibodies to OT-II mouse OVA-Mac-EVs, we observed that the intensity of antibody-detecting fluorescence increases together with FSC value, which refers to the size of the analyzed events (Figure 5B). This interesting observation led us to hypothesize that the antibody-induced enhancing effect may result from the aggregation of Mac-EVs. To investigate this hypothesis, we subjected OT-II mouse OVA-Mac-EVs that either had or had not been incubated with anti-OVA-323 antibodies to NTA. The mean size of both OVA-Mac-EV preparations was similar (i.e., 178.4 ± 2.8 nm for OVA-Mac-EVs non-incubated with antibodies and 176.2 ± 13.3 nm for antibody-incubated ones). However, in the case of the antibody-pre-incubated OVA-Mac-EVs, their size distribution seemed to shift towards the bigger particles, as suggested by the presence of particles of a size around 728 nm and 986 nm, which were not detected in antibody-non-incubated OVA-Mac-EVs (Figure 6A). Thus, at the next step we visualized both OVA-Mac-EV preparations with TEM microscope. This demonstrated that OVA-Mac-EVs that were incubated with antibodies had either separated or formed loose clusters, whereas the majority of the antibody-incubated OVA-Mac-EVs were tightly aggregated with one another (Figure 6B). These observations confirmed that treatment of the OVA-Mac-EVs with antigen-specific antibodies leads to their aggregation, which, in turn, is supposed to be responsible for antibody-induced enhancement of the suppressive activity of MHC class II-expressing Mac-EVs.

In addition, the binding of anti-OVA-323 antibodies to I-A/I-E^pos^ OVA-Mac-EVs from OT-II mice (Figure 5C) suggests that targeting of T cells may depend on specific interaction of TCR with an antigenic peptide complexed with MHC class II on Mac-EVs. If this is true, we assumed that binding of free antigenic peptide to TCR should interrupt this interaction and thus affect the suppressive activity of OVA-Mac-EVs. Thus, we pre-incubated OT-II mouse DTH effector T cells with OVA-323 peptide and then treated them with antibody-incubated or non-incubated OT-II mouse OVA-Mac-EVs prior to their adoptive transfer. Indeed, we observed that intact OVA-Mac-EVs failed to suppress DTH effector T cells pre-incubated with OVA-323 peptide, while anti-OVA-323 antibody-incubated OVA-Mac-EVs were strongly inhibitory (Figure 6C). Thus, we concluded that OVA-Mac-EV-bound antibodies could bind to OVA-323 peptide stuck in TCR, which enabled the targeting of DTH effector T cells (Figure 6D). In addition, the antibody-induced aggregation of OVA-Mac-EVs seems to increase the amount of EVs that target a particular effector T cell at the same time (Figure 6D).

## 3. Discussion

**Figure 7:**
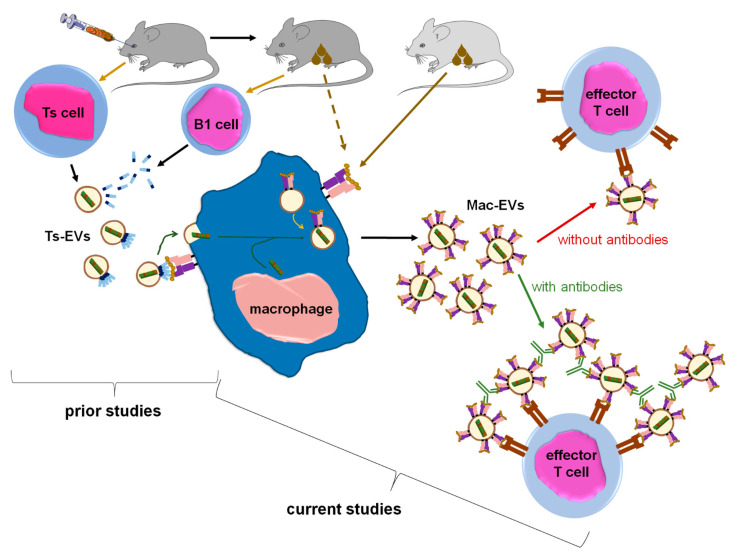
Summary of the findings in the context of existing knowledge. From prior studies we knew that intravenous tolerization of mice with antigen-coupled syngeneic red blood cells induces Ts cells to release miRNA-150 in Ts-EVs that are then coated with antigen-specific antibody light chains. The latter are secreted by B1 cells activated by antigen applied to mice via cutaneous route. Subsequently, Ts-EVs target macrophages that are able to present antigenic determinant complexed with MHC class II after cutaneous immunization of donor animal. In turn, Ts-EV-targeted macrophages suppress CHS and DTH responses in mice. Currently, we have shown that macrophages treated with Ts-EV-transmitted miRNA-150 begin to release Mac-EVs that also contain miRNA-150, which finally inhibits CHS and DTH effector T cell activity. Crucially, Mac-EVs express MHC class II molecules that are likely complexing the antigenic determinant, which enables the specific targeting of effector T cells, and also binding of specific IgG antibodies by Mac-EVs. The latter finding allowed us to discover that antigen-specific antibodies aggregate Mac-EVs, which greatly enhances their suppressive activity against effector T cells. Thus, one can conclude that macrophages multiply the number of miRNA-150 copies and release them in MHC class II-positive EVs to amplify the suppressive effect and direct it against specific effector T cells, and that this effect is significantly enhanced by incubating Mac-EVs with antigen-specific antibodies.

EVs are considered key players in intercellular signaling pathways that are able to specifically deliver the enclosed cargo to the desired target cells to modify their biological activities. The last two decades have brought about numerous discoveries that have greatly increased the knowledge of the mechanisms underlying these exceptional functions of EVs. Accordingly, our current research findings propose a novel approach to enhance EVs’ therapeutic potential, especially that EVs seem to have numerous advantages over synthetic liposomes and nanoparticles [19]. However, rapid clearance from circulation along with insufficient targeting of desired cells could limit their biological efficacy [3], and thus clinical usefulness [20]. Our current research findings suggest that these disadvantages could be overcome by aggregating homogenous EVs with antigen-specific antibodies (Figure 7).

Along these lines, after release by the parental cell, EVs seem to disperse quickly in body fluids where they mix with their counterparts from other cellular sources [21], and form a colloidal suspension with a natural tendency to aggregate [22]. On the one hand, EVs’ aggregation is considered an unwanted side effect that makes reliable single-vesicle profiling very difficult [23,24]. On the other hand, one can speculate that aggregation is a physiological mechanism, which evolved to increase EVs’ half-life in body fluids. However, such “colloid aggregates” are likely formed by morphologically and functionally variable EVs due to a very high heterogeneity of EV population that circulates within body fluids. In contrast, herein we observed that a cross-linking of EVs that express the same antigenic determinant with specific antibodies seems to produce homogeneous aggregates, characterized not only by increased half-life, but especially by augmented ability to target desired cells. This in turn greatly enhances their biological effectiveness in vivo, as observed in the current circumstances.

Our observations are in line with another report demonstrating that MHC class II-expressing EVs activated T cells less efficiently than their parental dendritic cells, unless EVs had been cross-linked with latex beads [25]. The antibody-mediated aggregation of EVs proposed herein seems to take an advantage over the latter approach, since the antibodies could be physiologically degraded in the targeted cells, in contrast to latex beads. Furthermore, EVs released into 48-h culture supernatant by OVA-loaded, lipopolysaccharide (LPS)-stimulated dendritic cells have been shown to bind antibodies present in sera of OVA-immunized mice, and to induce OVA-specific T-cell proliferation in vivo [26]. Thus, these observations support the hypothesis that EVs could bind antigen-specific antibodies, which potently enhances their activity.

From another point of view, the pattern of expressed markers differs between PCL-Mac-EVs and OVA-Mac-EVs, especially given that only the latter were found to be MHC class II-positive. This discrepancy may result from subtle, but meaningful, differences in the activation of APCs by hapten and protein antigen. The successful presentation of antigenic peptides derived from the latter requires its endocytosis and processing by APCs, which in turn become activated. In contrast, reactive hapten derivatives could covalently bind to APC membrane proteins [27]. As a consequence, such directly haptenated APCs [28] may not become primed enough [29] to be capable of translocating hapten/MHC class II complexes onto EVs’ membrane [30]. This seems to apply to current experimental conditions, in which OVA-Ts-EV-treated macrophages from OVA-immunized OT-II mice produced MHC class II-positive OVA-Mac-EVs, while TNP-Ts-EV-treated macrophages from PCL-sensitized CBA mice released PCL-Mac-EVs that failed to express MHC class II molecules. This assumption is additionally supported by the fact that, in contrast to OVA-Mac-EVs, the suppressive activity of PCL-Mac-EVs was not affected by incubation with anti-TNP IgG antibodies. Furthermore, one can speculate that an analogous mechanism may explain the lack of CD81 expression on PCL-Mac-EVs, since this tetraspanin was reported to be involved in both the activation of antigen-primed macrophages [31], and the trafficking of MHC class II molecules to EVs during their intracellular biogenesis [32].

On the other hand, both PCL- and OVA-Mac-EVs expressed a CD9 marker involved in sorting of MHC class II molecules into EVs [30], and likely playing a pivotal role in Evs’ trafficking and uptake by targeted cells [32]. Thus, in current conditions, we assumed that anti-CD9 monoclonal antibodies abolished the suppressive activity of PCL-Mac-Evs by impairing their docking to the membrane and further uptake by CHS effector T cells. It has been postulated that the fusogenic activity of CD9 depends on its association with different adhesion molecules [33], including lymphocyte function-associated antigen-1 (LFA-1) [34], on acceptor cells. Accordingly, the internalization of Evs by tumor cells has been recently found to be either impaired by treatment with antigen-binding fragment (Fab) of anti-CD9 antibody or slightly enhanced by incubation with anti-CD9 antibody [18]. The latter observation is contradictory to our findings. However, in those circumstances, Evs and tumor cells were incubated together in the presence of anti-CD9 antibodies or their derived Fab portions. This allowed simultaneous binding of CD9 on both Evs and acceptor cells, each by one of the antideterminants of the whole IgG antibody, which has been proposed to be responsible for the enhancement of Evs’ endocytosis [18,35]. In contrast, in our experiments, PCL-Mac-Evs were firstly incubated with anti-CD9 monoclonal antibodies and then ultracentrifuged prior to the treatment of the CHS effector cells, which favors the binding of the EV-membrane expressed CD9 by the majority of antibody antideterminants. Similarly, the incubation of OVA-Mac-Evs with anti-OVA-323 antibodies led to their aggregation. However, some of the antideterminants might remain unoccupied, and thus could bind to the OVA-323 peptides that were stuck to the TCR of the targeted effector T cells, thereby allowing DTH suppression.

Furthermore, the binding of anti-OVA-323 antibodies and the expression of I-A/I-E molecules by OVA-Mac-Evs from OT-II mice implies that they display OVA-323 peptides complexed with MHC class II. Thus, in turn, OVA-Mac-EVs can be considered capable of the specific targeting of DTH effector T cells by interacting with their TCR. This hypothesis is supported by the observation that OVA-induced DTH effector T cells from OT-II mice cannot be suppressed by OVA-Mac-EVs when pre-incubated with OVA-323 peptide. Hence, we suggest that the expression of antigenic peptides complexed with MHC allows addressing EVs to T cells with antigen-specific TCR (Figure 7). This in turn enables the highly specific and selective delivery of EV-transferred cargo, which has great importance in attempts to therapeutically suppress antigen-specific T cells.

On the other hand, MHC-expressing EVs are considered capable of antigen presentation [36,37]. However, previoys studies reported that EVs displaying peptides complexed with either MHC class I or MHC class II could not directly stimulate CD8+ cytotoxic T cells [38] or CD4+ helper T cells [25], respectively. Instead, for the efficient stimulation of T cells, these EVs had to be uptaken by mature dendritic cells [25,38]. Simultaneously, it was suggested that in such conditions EV-targeted mature dendritic cells provide costimulatory signal from CD80/CD86 molecules, which enables efficient priming of naive T lymphocytes [39]. Conversely, artificially generated nanoparticles carrying MHC/peptide complexes but not costimulatory molecules have been recently proposed for therapeutic activation of immune tolerance owing to T-cell anergy [40]. Accordingly, our initial analysis demonstrated that OVA-Mac-EVs do not express CD80 and CD86 costimulatory molecules (data not shown). Thus, one can speculate that OVA-Mac-EVs could induce the anergy of antigen-specific T cells by presenting OVA-323 peptide in the absence of costimulatory signals. Herein, this effect seems to reinforce the miRNA-150-mediated tolerogenic potential of OVA-Mac-EVs.

Thus far, miRNA-150 has been unequivocally proven to mediate the suppressive and self-tolerogenic potential of Ts-EVs [5,13]. Remarkably, the current research findings demonstrated that suppressive activity of Mac-EVs depends on miRNA-150 as well (Figure 7). Thus, we assumed that antigen-presenting macrophages play a pivotal role in miRNA-150-induced immune suppression by transferring the multiplied suppressive signal from a few Ts cells to numerous effector T cells in an antigen-specific manner. In this regard, macrophages could be compared to a “resonance tube” (Figure 7). Interestingly, a similar function could likely be applied to dendritic cells, which were suggested to amplify both antigen-presentation and maturation processes by transferring, respectively, peptide/MHC complex-bearing EVs [38], and EV-contained miRNAs [41], to neighboring dendritic cells. It is also worth noting that recent research demonstrated the significant modification of dendritic cell function, cytokine production especially, under the influence of the regulatory T cell-derived EVs. In that study, one of the miRNA molecules carried by regulatory T cell EVs and suspected to play a crucial role in the activation of the tolerogenic phenotype of dendritic cells was miRNA-150 [14]. Thus, miRNA-150 could likely be considered a general inducer of the APC tolerogenic phenotype.

Interestingly, the reduced expression of miRNA-150 in ear skin samples was detected in mice with elicited CHS response to dinitrofluorobenzene hapten [42], bringing another piece of evidence for the involvement of miRNA-150 in the regulation of CHS. Our previous studies showed that the suppressive activity of Ts-EV-targeted macrophages is associated with increased apoptosis as well as with impaired activation and proliferation of antigen-specific effector T cells [8,13]. The current observations confirmed that these effects are induced in targeted CD4+ T cells by Mac-EV-transferred miRNA-150. The downregulatory activity of miRNA-150 has already been shown in immune tolerance to transplanted antigens, and was similarly associated with an inhibited activation and proliferation as well as an increased apoptosis of CD4+ T lymphocytes [43]. Our previous research also suggested that miRNA-150 decreases T-cell reactivity to IL-2 [5,7]. Other studies revealed that miRNA-150-induced and IL-2-mediated signaling cascades in T cells are mutually dependent [44,45,46], and thus may underlie the Mac-EV-induced effects in DTH effector T cells. However, this aspect requires further investigation. Similarly, further studies are needed to specify the molecular targets of miRNA-150 in both macrophages and DTH effector T cells.

The homology of the sequences of mouse and human miRNA-150 allowed us to subject OVA-Ts-EVs to the standardized model for testing of EV-mediated intercellular interactions at the immune synapse. With this model, the unidirectional transfer of miRNA-loaded, CD63^pos^ EVs from intact Jurkat T cells to SEE-pulsed Raji B cells was formerly reported [17]. Similarly to that report, CD81-GFP-transfected Raji B cells displayed almost no vesicle transfer to the Jurkat T cells after SEE-stimulation in current conditions. However, and surprisingly, the treatment of CD81-GFP-transfected Raji B cells with OVA-Ts-EVs induced the SEE-promoted transmission of CD81-GFP^pos^ vesicles to Jurkat T cells. This implies that Ts-EV-treated APCs may suppress effector T cells by transferring EV-enclosed regulatory miRNA-150 at the immune synapse. Additionally, we observed the accumulation of CD81 in Ts-EV-treated, SEE-stimulated Raji B cells close to the site of CD3 accumulation in Jurkat T cells. Other studies demonstrated that CD81 in APCs physiologically co-localize with T cell-expressed CD3 at the cell–cell contact area to support immune synapse formation during antigen presentation [47]. However, after 24 h of co-culture, we found some Jurkat T cells that emitted CD81-GFP-dependent fluorescence, which seems to confirm that T cells could be targeted by CD81^pos^ EVs from Ts-EV-treated APCs at the immune synapse. However, these interactions remain a subject of further investigation.

Further evidence for EV-mediated signaling at the immune synapse was provided by a previous study demonstrating an alternative pathway for MHC class II sorting in antigen-loaded dendritic cells. Namely, interaction with antigen-specific CD4+ T cells induces in dendritic cells the generation of multivesicular bodies containing luminal vesicles displaying MHC class II and CD9, which are then released as EVs and transferred to these T cells [30]. Our current results show that such EV-mediated signaling at the immune synapse could be used for the transfer of regulatory miRNA-150, and thus may ensure the antigen-specificity of the induced immune tolerance.

Along these lines, Ts-EVs are surface coated with antigen-specific antibody light chains that confer the specificity of Ts-EVs’ suppressive activity [12]. Recent findings allowed us to conclude that OVA-Ts-EV-coating antibody light chains bind antigenic peptides complexed with MHC on APCs [9,21]. This enables the antigen-specific delivery of miRNA-150 to APCs, which in turn become tolerogenic. The current research showed that OVA-Ts-EV-targeted APCs release miRNA-150-carrying OVA-Mac-EVs that could be equipped with antigenic determinant complexed with MHC class II, which allows OVA-Mac-EVs to target OVA-specific T cells, likely by binding to their TCR. Thus, such a circuit allows us to maintain the antigen-specificity of EV-miRNA-150-mediated immune suppression at each of its steps. Altogether, one can conclude that EVs are naturally predisposed to specifically target desired cells to deliver selected cargo [48].

To summarize, after discovering of the role of miRNA-150 in an antigen-specific suppression of mouse CHS and DTH responses [5,9], we demonstrated that Ts-EVs target antigen-presenting macrophages [8]. Our current results show that Ts-EV-treated macrophages release Mac-EV-enclosed miRNA-150 to suppress effector T cells. Immune synapse formation can trigger the release of Mac-EVs by miRNA-150-targeted APCs. PCL-Mac-EVs target CHS effector T cells in a CD9-dependent manner, while DTH effector T cell-suppressing OVA-Mac-EVs express MHC class II and thus can bind OVA-specific antibodies, which increases their suppressive activity in vivo. The latter observation led to a unique discovery of the antibody-induced aggregation of MHC class II-positive EVs that enhances their therapeutic potential in the treatment of the active DTH response. To the best of our knowledge, this is the first demonstration of the enhancement of EVs’ biological activity due to their aggregation with antigen-specific antibodies.

## 4. Materials and Methods

### 4.1. Antigens, Antibodies, Reagents and Culture Media

Following antigens, haptens and antibodies were used: ovalbumin (OVA), OVA 323-339 peptide (OVA-323, Sigma, St Louis, MO, USA); purified and biotinylated rabbit polyclonal anti-OVA-323-339 IgG antibodies, fluorescein isothiocyanate (FITC)-conjugated rabbit polyclonal IgG isotype antibodies (Innovagen, Lund, Sweden); purified mouse anti-trinitrophenol (TNP) IgG1 monoclonal antibody of A111-3 clone, purified and phycoerythrin (PE)-conjugated rat anti-mouse CD9 monoclonal antibody (clone KMC8), PE-conjugated rat anti-mouse CD63 monoclonal antibody (clone NVG-2), PE-conjugated hamster anti-mouse CD81 monoclonal antibody (clone Eat2), PerCP-Cy- or PE-conjugated rat anti-mouse I-A/I-E monoclonal antibody of M5/114.15.2 clone, FITC- or horseradish peroxidase (HRP)-conjugated streptavidin (all from BD Biosciences, San Diego, CA, USA); PCL (TNP-Cl, Chemtronix, Swannanoa, NC, USA); trinitrobenzene sulphonic acid (TNBSA, Eastman Chemicals, Rochester, NY, USA).

Following reagents and media were used: aldehyde/sulfate latex beads 4% *w*/*v*, 4 µm (cat. No A37304, Life Technologies, ThermoFisher Scientific, Carlsbad, CA, USA); mineral oil heavy fraction, protein-free Mishell–Dutton medium, RPMI 1640, minimal essential medium with amino acids, HEPES, cacodylic buffer, 2-mercaptoethanol, phenol-chloroform mixture (Sigma, St Louis, MO, USA); Dulbecco’s phosphate-buffered saline (DPBS), penicillin/streptomycin, sodium pyruvate, L-glutamine (Gibco Life Technologies, Grand Island, NY, USA); acetone, ethanol, glucose (P.O.Ch., Gliwice, Poland); 1-ethyl-3-(3-dimethylaminopropyl)carbodiimide (EDC, Pierce, ThermoFisher Scientific, Waltham, MA, USA); ethylenediaminetetraacetic acid (EDTA, BDH, Poole, UK); extra virgin olive oil (Basso Fedelee Figli, San Michele di Serino, Italy); 3,3′,5,5′-tetramethylbenzidine (TMB) liquid substrate for enzyme-linked immunosorbent assays (ELISA, BD Biosciences, San Diego, CA, USA).

### 4.2. Mice

Ten- to fourteen-week old mice of the C57BL/6 and miRNA-150^−/−^ knock-out inbred strains were from Jackson Laboratories (Bar Harbor, ME) and CBA mice were either from Jackson Laboratories or, together with OT-II T-cell receptor (TCR) transgenic mice and some of C57BL/6 mice, from the 2nd Breeding Unit of the Jagiellonian University Medical College, Faculty of Medicine (Krakow, Poland). Mice were fed autoclaved food and water ad libitum.

### 4.3. Induction of Tolerance and Harvest of Suppressor T Cell-Derived EVs (Ts-EVs)

Freshly collected, pelleted mouse erythrocytes were conjugated with TNP hapten by mixing with TNBSA solution in cacodylic buffer (5.7 mg/mL) in a ratio of 7 mL of TNBSA solution per 1 mL of erythrocytes, and incubating 20 min at room temperature, in darkness on hematological roller. Otherwise, mouse erythrocytes in 50% DPBS suspension were labeled with OVA protein by incubation with 1% OVA solution in DPBS (in a ratio 1:5 *v*/*v*) for 1 h at room temperature on hematological roller in the presence of EDC used as a coupling activating agent [9,12]. Ts cell-mediated tolerance was induced in mice as described previously [5,8]. In brief, CBA, miRNA-150^−/−^ or C57BL/6 mice were intravenously injected with 0.2 mL of 10% DPBS suspension of either TNP-coupled or OVA-coupled syngeneic erythrocytes on days 0 and 4, which, on day 9, was followed by, respectively, contact sensitization on shaved abdominal skin with 0.15 mL of 5% PCL solution in ethanol:acetone (3:1 *v*/*v*) [5,8], or by double intradermal immunization with 0.2 mL of 0.5 mg/mL OVA-saline solution on day 8 and 9 [9,49]. On day 11 spleens and lymph nodes containing activated Ts cells were collected from tolerized mice and single cell suspensions were cultured in protein-free Mishell–Dutton medium at a concentration of 2 × 10^7^ cells/mL for 48 h [5,9,10]. The resulting supernatant was subsequently centrifuged at 300 *g* and 3000 *g* for 10 min, filtered through 0.45 µm and 0.22 µm molecular filters and then ultracentrifuged twice at 100,000 *g* for 70 min at 4 °C [5,8,9]. Resulting pellet was resuspended in DPBS [5], and used as either TNP-Ts-EVs or OVA-Ts-EVs, respectively [5,9].

Pellets of non-suppressive, control vesicles, so-called negative factor [5,7,8], were obtained by ultracentrifugation of supernatants of 48-h culture of lymph node and spleen cells collected from naive mice, and processed as above.

In some instances, mixtures of DNA/RNA were extracted with phenol-chloroform from culture supernatants containing either Ts-EVs or control, non-suppressive vesicles (negative factor) by modified Chomczynski method [50], as described previously [7]. Where indicated, DNA/RNA extract was further treated with either DNase (D7231) or RNase A (R4375, both from Sigma, St Louis, MO, USA), or incubated with anti-miR-150 (Dharmacon RNAi Technologies, Lafayette, CO, USA), as described previously [7]. OVA-Ts-EVs were similarly incubated with anti-miR-150 (Dharmacon RNAi Technologies, Lafayette, CO, USA) [9].

### 4.4. Harvest of Macrophages and Their Treatment to Obtain Macrophage-Derived EVs (Mac-EVs)

Macrophage-enriched peritoneal exudate was induced by intraperitoneal injection of 1 mL of mineral oil into either naive, PCL-sensitized or OVA-immunized mice of CBA, C57BL/6, OT-II or miRNA-150^−/−^ strains [8]. Five days later, macrophage-containing exudate was harvested by washing the peritoneal cavity with ice-cold DPBS with heparin (5 U/mL). The percentage of non-specific esterase positive macrophages in exudates in each case exceeded 95% [51]. After washing, macrophages were treated for 30 min in 37 °C water-bath with DPBS-suspension of Ts-EVs, in a dose of approximately 1 × 10^9^ Ts-EVs per 1 × 10^6^ macrophages, as estimated by NTA [5]. In some instances, macrophages were similarly treated with phenol-chloroform extracts (PCE) of Ts-EVs or negative factor vesicles in a dose of about 3 µg of PCE nucleic acid mixture per 1 × 10^6^ cells [7]. After washing out of excessive vesicles or nucleic acids at 300 *g,* Ts-EV/PCE-treated macrophages were cultured at 37 °C and 5% CO_2_ in protein-free Mishell–Dutton medium at a concentration of 3 × 10^6^ cells/mL for 48 h, unless otherwise indicated in particular experiment. The resulting supernatant was subsequently centrifuged at 300 *g* and 3000 *g* for 10 min, filtered through 0.45 µm and 0.22 µm molecular filters and then ultracentrifuged twice at 100,000 *g* for 70 min at 4 °C. The resulting pellet containing Mac-EVs was resuspended in DPBS for experimental usage. In some experiments supernatants above the pellets, remaining after ultracentrifugation of macrophage culture supernatants, were also used for treatment of adoptively transferred effector cells. Mac-EVs pelleted by ultracentrifugation of supernatant from the culture of TNP-Ts-EV-pretreated macrophages from mice epicutaneously sensitized with PCL were termed PCL-Mac-EVs. Otherwise, Mac-EVs pelleted by ultracentrifugation of supernatant from the culture of OVA-Ts-EV-pretreated macrophages from mice intradermally immunized with OVA were termed OVA-Mac-EVs.

Where indicated, OT-II mouse OVA-Mac-EVs were incubated with anti-miR-150 (Dharmacon RNAi Technologies, Lafayette, CO, USA) for 2 h in 37 °C water-bath, and, after washing, were used to treat OT-II mouse DTH effector cells prior to their adoptive transfer to naive wild type C57BL/6 mouse recipients.

Macrophages and Mac-EVs were also subjected to miRNA deep-sequencing as described in Supplementary Methods section.

### 4.5. Treatment of Mac-EVs with Antibodies

PCL-Mac-EVs were incubated overnight on ice with purified anti-TNP IgG or anti-CD9 monoclonal antibodies in a dose of 6 µg per 1 × 10^10^ Mac-EVs, which was followed by ultracentrifugation to remove the unbound antibodies. Pellet was then used to treat CHS effector T cells, as described below. Similarly, OVA-Mac-EVs were incubated overnight on ice with purified or biotinylated anti-OVA-323 IgG antibodies or with FITC-conjugated isotype IgG antibodies in a dose of 6 µg per 1 × 10^10^ Mac-EVs, which was followed by ultracentrifugation to remove the unbound antibodies. Then, pellet was either analyzed in NTA (Nanosight), visualized with TEM microscope, coupled onto latex beads and analyzed cytometrically, or used for treatment of actively immunized mice or DTH effector T cells, as described below.

### 4.6. Adoptive Transfer of CHS or DTH Effector Cells

CBA mice were contact sensitized with 0.15 mL of 5% PCL solution in ethanol:acetone mixture (3:1 *v*/*v*) on shaved abdominal skin. Five days later lymph nodes and spleens were harvested from sensitized animals and proceeded for obtaining the single cell-suspension of CHS effector cells, which were then treated with PCL-Mac-EVs or control factors for 30 min in 37 °C water-bath and, after washing at 300 *g*, were transferred intravenously into naive recipients (7 × 10^7^ cells per mouse) that were immediately challenged to elicit CHS reaction by application of 10 µL of 0.4% PCL solution in acetone:olive oil (1:1 *v*/*v*) on each side of both ears. Twenty four hours later CHS reaction was measured as an increase in ear thickness (ear swelling response) with engineer’s micrometer (Mitutoyo, Japan) by blinded observer [5,8]. This method of CHS reaction assessment is essentially non-invasive, allows for repetition of measurements and obtained results strongly correlated with other methods [52].

CBA, OT-II or C57BL/6 mouse donors of DTH effector cells were immunized on two consecutive days (0 and 1) with OVA protein antigen (without an adjuvant) by intradermal injections into four sites of abdominal skin of 0.2 mL, in total, of a 0.5 mg/mL OVA solution in 0.9% NaCl [9,49]. On day 5 lymph nodes and spleens were harvested from immunized mice and obtained DTH effector cells were then treated with OVA-Mac-EVs or control factors as describe above, before intravenous transfer into naive recipients of wild type strain (7 × 10^7^ cells per mouse). Twenty four hours later recipient mice were injected intradermally into both ears with 10 µL of a 0.5 mg/mL OVA solution in 0.9% NaCl (challenge) to elicit DTH reaction, measured 24 h later, as described above. Where indicated, nylon wool non-adherent CHS or DTH effector T lymphocytes were isolated by triple separation of lymph node and spleen single-cell suspensions on nylon wool column [53], with recovery in a range of 75% for CHS effector T cells and 40% for DTH effector T cells. Then, effector T lymphocytes were treated with pelleted Mac-EVs pretreated with antibodies, as described below. DTH effector T lymphocytes from OT-II mice, prior to incubation with OVA-Mac-EVs, were incubated with OVA-323 peptide (7.5 µg/10^7^ T cells) for 20 min in 37 °C water-bath.

### 4.7. Active Immunization

C57BL/6 were immunized on two consecutive days (0 and 1) with OVA without an adjuvant, as described above. On day 5, mice were injected intradermally in both ears with 10 µL of a 0.5 mg/mL OVA solution in 0.9% NaCl to elicit DTH reaction, measured up to 120 h after challenge. After measuring of 24-h ear swelling, mice were injected intraperitoneally with either OVA-Mac-EVs alone or OVA-Mac-EVs preincubated with anti-OVA-323 antibodies (1 × 10^10^ OT-II mouse OVA-Mac-EVs in 0.2 mL DPBS per mouse), or with equivalent volume of DPBS in positive control group.

### 4.8. In Vitro Testing of Immune Synapse Formation

The human Jurkat T-cell line (E6.1 clone) and the lymphoblastoid Raji B-cell line (Burkitt lymphoma, both acquired from the DSMZ Organization, Braunschweig, Germany, and routinely tested for mycoplasma) were cultured in RPMI 1640 medium supplemented with GlutaMAX, HEPES (Gibco Life Technologies, Grand Island, NY, USA) and 10% fetal bovine serum (FBS, Hyclone, ThermoFisher, Waltham, MA, USA). At least 24 h prior to experimental culturing, cells were moved to medium supplemented with 10% FBS obtained from stock FBS that had been previously ultracentrifuged overnight at 100,000 *g* to remove its own vesicles. Raji B cells (15 × 10^6^ cells) resuspended in Opti-MEM medium (Gibco Life Technologies, Grand Island, NY, USA) were transiently transfected with 25 μg of CD81-GFP DNA plasmids by electroporation with the gene-pulser III system from Bio-Rad Laboratories (240 V, 975 mΩ, 27 ms) in 4 mm cuvettes (Bio-Rad, Hercules, CA, USA), as described elsewhere [17,47,54]. Cells were then cultured in FBS-supplemented RPMI 1640 medium containing 2 mg/mL of G418 antibiotic (Invitrogen, Carlsbad, CA, USA) and the efficacy of electroporation and CD81-GFP plasmid incorporation was assessed cytometrically 24 h later.

For flow cytometric analysis of cells after formation of conjugates, CD81-GFP-transfected Raji B cells were pulsed with Staphylococcal enterotoxin type E (SEE) superantigen (0.5 µg/mL) for 30 min at 37 °C and, after washing, were cultured with Jurkat T cells for 24 h in the presence of OVA-Ts-EVs. Afterwards, cells were stained with fluoresceinated antibodies against CD19 (also allowing to distinguish CD19^neg^ T cells). A viability staining was performed as well with Ghost Dye V510 (TONBO Biosciences, San Diego, CA, USA). Cells were analyzed in a FACS Canto II (BD Bioscience, San Jose, CA, USA) and then FCS files were analyzed in FlowJo software [55].

For immune synapse formation assay evaluated in fluorescence confocal microscopy, Jurkat T cells were loaded with the CMAC cell tracker. CD81-GFP-transfected Raji B cells were treated with OVA-Ts-EVs for 4 h at 37 °C, which was followed by pulsing with SEE (0.5 µg/mL) for 30 min at 37 °C. Afterwards, Jurkat T cells (1 × 10^5^ cells) were mixed with the CD81-GFP-transfected, Ts-EV-pretreated Raji B cells (in a ratio 1:1) and plated onto Poly-L-Lys-coated slides for 1 h incubation at 37 °C, or were standardly cultured for 24 h on 96-well culture plates and then moved onto Poly-L-Lys-coated slides and incubated for 30 min at 37 °C. Then, cells were fixed with 4% paraformaldehyde and 0.12 mM sucrose in PHEM buffer and permeabilized at room temperature with 0.2% Triton X-100 in immunofluorescence solution. After blocking with immunofluorescence solution, cells were stained with selected primary and then secondary antibodies. Finally, cells were mounted on Prolong Gold and analyzed with a Leica SP5 confocal microscope (Leica, Wetzlar, Germany) fitted with a HCX PL APO×63/1.40–0.6 oil objective and images were processed and assembled using Image J software [54].

### 4.9. Cytometric Analysis of Mac-EVs 

Aldehyde/sulfate latex beads, washed and resuspended in DPBS, were incubated with DPBS-suspension of either PCL-Mac-EVs or OVA-Mac-EVs (in a ratio about 1 × 10^6^ beads per 1 × 10^9^ vesicles, as estimated by NTA) in a total volume of 1 mL of DPBS for 10 min at room temperature with gentle agitation [56,57,58]. Afterwards, 1 mL of DPBS was added and samples were incubated for next 2 h at room temperature with gentle agitation. After addition of 1 mL of 100 mM glycine solution for blocking of remaining binding sites, samples were incubated for 30 min at room temperature with gentle agitation. Then, beads coated with Mac-EVs were washed thrice in DPBS with 0.1% bovine serum albumin (BSA) by centrifugation at 600 *g* for 10 min. After resuspending in DPBS, vesicle-coated beads were incubated with PE-conjugated anti-mouse CD9, CD63, CD81 or I-A/I-E (MHC class II) monoclonal antibodies for 40 min at room temperature in darkness, which was followed by triple washing with DPBS containing 0.1% BSA. In some instances, beads coated with OVA-Mac-EVs, pre-incubated overnight with biotinylated anti-OVA-323 IgG antibodies, were stained with streptavidin-FITC and, where indicated, with PE-conjugated anti-CD9 and PerCP-Cy-conjugated I-A/I-E monoclonal antibodies. Latex beads coated with OVA-Mac-EVs, pre-incubated overnight with FITC-conjugated isotype IgG antibodies, were only stained with PE-conjugated anti-CD9 and PerCP-Cy-conjugated I-A/I-E monoclonal antibodies. Otherwise, beads coated with OVA-Mac-EVs were stained with biotinylated anti-OVA-323 IgG antibodies for 1 h and then with streptavidin-FITC for 40 min at room temperature. After washing, DPBS-resuspended, vesicle-coated beads were acquired by a BD FACSCalibur and data were analyzed using BD CellQuest Pro software (BD Bioscience, San Jose, CA, USA).

### 4.10. NTA and TEM Analysis of Mac-EVs

OT-II mouse OVA-Mac-EVs that were incubated overnight on ice either alone or with anti-OVA-323 antibodies, after dilution with filtered DPBS, were subjected to NTA by an experienced observer unaware of experimental protocol and samples [5]. Furthermore, OT-II OVA-Mac-EVs, similarly incubated with or without antibodies, were absorbed onto cupper grid and negatively stained with 3% uranyl acetate. Then, samples were visualized with TEM microscope (JEOL 2100HT, Tokyo, Japan), equipped with TVIPS camera, and analyzed with EMMENU 4 software by an experienced observer unaware of experimental protocol and samples.

### 4.11. Assessing of Binding of Antibodies to OVA-Mac-EVs by ELISA

Ninety six-well ELISA plates (Corning, NY, USA) were coated with purified anti-mouse CD9 antibody (4 µg/mL) diluted in sodium carbonate buffer (pH 9.5), by incubation overnight at 4 °C. After washing, blocking the plate wells with 2% BSA in PBS for 2 h at room temperature, and repeated washing, 50 µL/well of OT-II mouse OVA-Mac-EVs (approximately 1 × 10^8^ EVs/well) in PBS was pipetted to selected wells, and the plate was incubated for 2 h at room temperature. Then, 50 µL/well of biotinylated anti-OVA-323 IgG or biotinylated isotype IgG diluted in PBS (1 µg/mL) was added to selected wells, and the plate was incubated overnight at 4 °C. After extensive washing, 100 µL/well of streptavidin-HRP (diluted 1:250 in PBS with 0.1% BSA) was added to each well, which was followed by incubation for 45 min at room temperature and extensive washing. Finally, 100 µL/well of TMB substrate was pipetted to each well and the reaction was stopped after 5 min by adding 50 µL/well of 1 M H_3_PO_4_. The absorbance was measured at 450 nm with a reference wavelength of 570 nm. Yielded absorbance values were blanked by subtracting the values measured in respective wells without added OVA-Mac-EVs and then normalized to the mean absorbance value detected in wells with added OVA-Mac-EVs but not biotinylated antibodies.

### 4.12. Statistics

In vivo experiments were carried out 2–3 times and representative results were shown in the figures, prepared with the use of GraphPad Prism 8, and Adobe Photoshop 22.4 software. All groups consisted of 5 mice and average value of nonspecific increase in ear thickness caused by chemical irritation by hapten or protein solution in challenged but not immunized mouse littermates was subtracted from average values in experimental groups to obtain net swelling value (*delta*), expressed as average *delta* ± standard error of the estimate of mean value (SEM). Statistical significance of the data was estimated (after control of meeting of test assumptions) in one-way Analysis of Variance (ANOVA) with post hoc RIR Tukey test with the use of STATISTICA.10 or GraphPad Prism 8 software, and *p* < 0.05 was considered statistically significant. In vitro experiments were performed at least twice and data were analyzed either with one-way or two-way ANOVA or with two-tailed Student *t*-test, while miRNA deep-sequencing was performed once, and heatmaps were drawn with GraphPad Prism 8.

### 4.13. Study Approval

All animal experiments were performed in accordance with the principles of the Basel Declaration and ARRIVE guidelines, and were approved by Ethics Committees of both Yale (approval number 07381) and Jagiellonian (approvals number 39/2011, 154/2013, 51/2017 and 433/2020) Universities.

## 5. Conclusions

We observed that macrophage-induced suppression of DTH is mediated by their EVs, which carry miRNA-150, and appears to be released more efficiently after immune synapse formation. Moreover, the activity of MHC class II-expressing Mac-EVs was found to be significantly enhanced by pre-incubation with antigen-specific antibodies. The detailed analysis of this phenomenon provided the main discovery of the current study, i.e., the first-time demonstration that the antigen-specific antibodies enhance the tolerogenic activity of MHC class II-expressing EVs by promoting their aggregation.

Therefore, our findings have a substantial translational potential in a field of clinically applicable methods for both the induction of antigen-specific immune tolerance and increasing the in vivo therapeutic activity of EVs.

## 6. Patents

K.N. and K.B. declare that they are inventors in a patent application number P.435582 submitted by Jagiellonian University, Krakow, Poland for the method of generating the antibody-aggregated macrophage EVs.

## Data Availability

The data presented in this study are contained within the article.

## Supplementary Materials

The following are available online at https://www.mdpi.com/article/10.3390/ph14080734/s1, Supplementary Methods: Deep-sequencing of miRNAs in Mac-EVs and macrophages, Supplementary references, Figure S1: PCA plot, Figure S2: Inhibiting miRNA-99a with an antagomiR failed to significantly alter the activity of OVA-Mac-EVs.
