# Supplementary material for: Antibodies Enhance the Suppressive Activity of Extracellular Vesicles in Mouse Delayed-Type Hypersensitivity
Source: Pharmaceuticals (Basel). 2021 Jul 27;14(8):734. doi: 10.3390/ph14080734 (PMC8398949; doi:10.3390/ph14080734)
Supplement: Supplementary file 1 [file pharmaceuticals-14-00734-s001.zip › pharmaceuticals-1290398-supplementary.pdf]

Supplementary Materials

Katarzyna Nazimek et al.

Antibodies enhance miRNA-150-dependent suppressive activity of MHC class II-positive extracellular vesicles in mouse delayed-type hypersensitivity

Supplementary Methods with references………………………………………… p. 2-3

Figure S1……………..………………………………………………..………… p. 4

Figure S2……………..………………………………………………..………… p. 5

Supplementary Methods

Deep-sequencing of miRNAs in Mac-EVs and macrophages

Macrophages from OVA-immunized C57BL/6 mice were treated with OVA-Ts-EVs for 30 minutes in 37°C water-bath, then washed and cultured in protein-free medium. Macrophages and supernatants were collected after 5 minutes, 24 hours and 48 hours of culture, and then cells together with pelleted OVA-Mac-EVs were subjected to deep-sequencing of miRNAs. For this purpose, total RNA was extracted with the use of miRCURY RNA Isolation Kit (Exiqon, Vedbaek Denmark), according to the manufacturer's protocol. Sequencing libraries were then prepared with NEBNext Multiplex Small RNA Library Prep for Illumina (New England Biolabs, Ipswich, MA). After ligation of the 5' and 3′ adapters, constructs were reverse transcribed and amplified in minimal number of polymerase chain reaction (PCR) cycles, which indexed products were purified on spin columns (Qiagen, Hilden, Germany) and eluted in water. Resulting small-RNA libraries were controlled for quality on D1000 ScreenTape and TapeStation device (Agilent, Santa Clara, CA) and size-selected for fragments around 146 bp in length, corresponding to adapter ligated and indexed miRNAs with removal of the side products, by automated gel electrophoresis BluePippin system (Sage Science, Beverly, MA) on dye-free cassettes containing 3% agarose gel with internal marker. After fluorometric measurement of concentration on Quantus (Promega, Fitchburg, WI), it was normalized to 10 pM final concentration on flow cell, and sequencing libraries were spiked-in with 1% phiX internal control. Finally, sequencing was performed on MiSeq instrument (Illumina, San Diego, CA) using v3 reagents.

RNA-sequencing data pre-processing and filtering

The quality control of each RNA-sequencing was performed using the FastQC software (Babraham Bioinformatics). After filtering out of low-quality sequences and adapter trimming, all reads shorter than 16 bp were removed, the remaining sequences were mapped to the GRCm38/mm10 genome and annotated to the mouse GENCODE M11 file using STAR aligner [1].

Statistical analysis of RNA-sequencing data

RNA-sequencing associated statistical analysis was performed using the statistical environment R (cran.r-project.org). Raw reads aligned to specific RNAs were normalized according to the pipeline implemented in the 'edgeR' package. The resulting pseudo-counts were analyzed using custom code (available upon request). The Principal Component Analysis (PCA) was performed by means of the 'prcomp'

function. For the purpose of exploratory statistical analysis of miRNA expression profiles, the entropy and coefficient of variation (i.e. standard deviation divided by the mean) was calculated using custom functions, and subsequently outliers were detected using the chi-square test as implemented in the package 'outlier' (function 'chisq.out.test'). Note that entropy was used as a measure of stability of expression profiles in previous work [2,3]. In this pilot analysis related to the detection of RNAs with highly variable expression profiles, a relaxed threshold of statistical significance, set to 0.1, was applied (this is due to small sample size). Since, in this analysis of outliers, only one RNA specific to EV samples was identified, we used a similarity index-based analysis (I-index, as described in [4,5]) to detect additional miRNAs with variable expression profiles specific to EV samples. The Gene Ontology (GO) enrichment analysis was performed as implemented in package 'miRNAtap'. Briefly, for each miRNA of interest predicted target mRNAs were identified and subsequently, on this set of mRNAs, the classical enrichment analysis (based on the Kolmogorov-Smirnov test) was performed.

Supplementary references

1. Dobin, A.; Gingeras, T.R. Mapping RNA-seq Reads with STAR. *Curr Protoc Bioinformatics* **2015**, *51*, 11.14.1-11.14.19, doi:10.1002/0471250953.bi1114s51.
2. Mohanapriya, S.; Elavarasi, S.A.; Akilandeswari, J. Clustering Gene Expression Data Using Shannon's Entropy. In Proceedings of the 2011 International Conference on Recent Trends in Information Technology (ICRTIT); IEEE: Chennai, India, June 2011; pp. 1116–1120.
3. Webb, A.; Papp, A.C.; Curtis, A.; Newman, L.C.; Pietrzak, M.; Seweryn, M.; Handelman, S.K.; Rempala, G.A.; Wang, D.; Graziosa, E.; et al. RNA Sequencing of Transcriptomes in Human Brain Regions: Protein-Coding and Non-Coding RNAs, Isoforms and Alleles. *BMC Genomics* **2015**, *16*, 990, doi:10.1186/s12864-015-2207-8.
4. Cebula, A.; Seweryn, M.; Rempala, G.A.; Pabla, S.S.; McIndoe, R.A.; Denning, T.L.; Bry, L.; Kraj, P.; Kisielow, P.; Ignatowicz, L. Thymus-Derived Regulatory T Cells Contribute to Tolerance to Commensal Microbiota. *Nature* **2013**, *497*, 258–262, doi:10.1038/nature12079.
5. Rempala, G.A.; Seweryn, M. Methods for Diversity and Overlap Analysis in T-Cell Receptor Populations. *J Math Biol* **2013**, *67*, 1339–1368, doi:10.1007/s00285-012-0589-7.

Figure S1

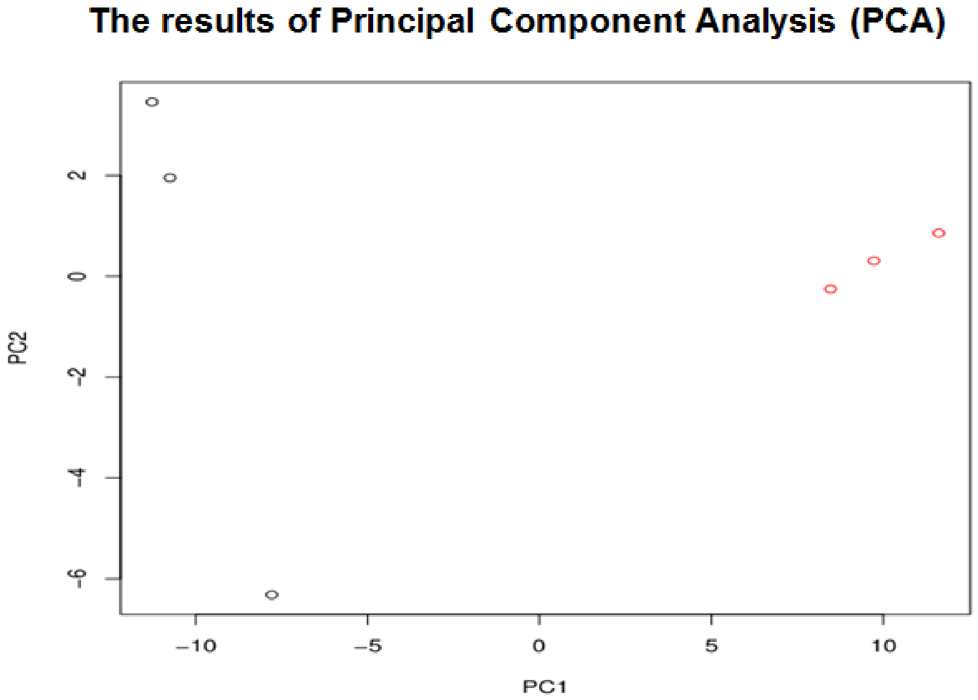

The presented PCA-plot is based on the standard Principal Component Analysis in R environment. The red dots represent the samples from Mac-EVs and the black dots the samples from Ts-EV-pretreated macrophages. The first Principal Component is on the horizontal and the second on the vertical axis.

Figure S2

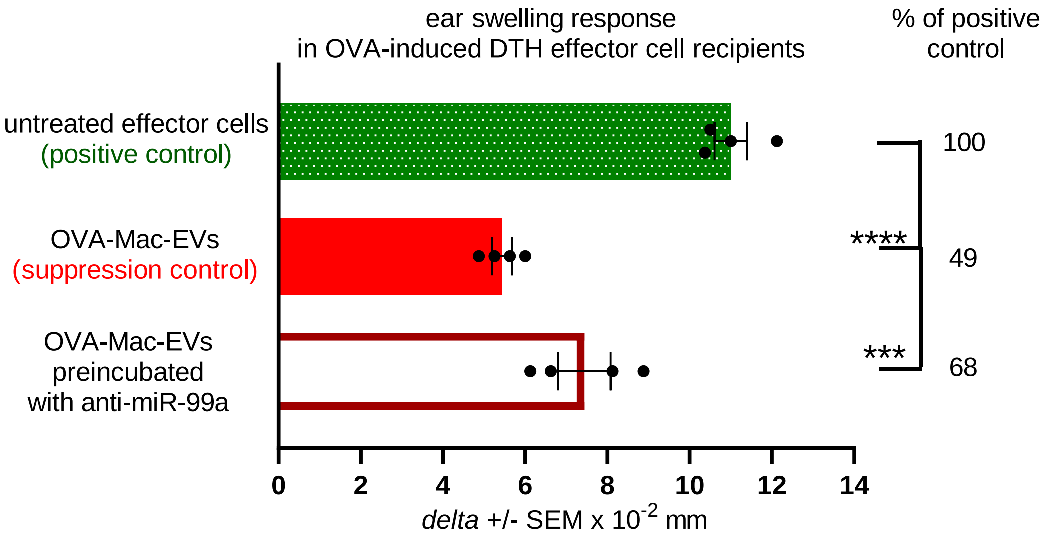

Figure S2. Inhibiting miRNA-99a with an antagomiR failed to significantly alter the activity of OVA-Mac-EVs. DTH effector cells were incubated with OVA-Mac-EVs, where indicated pretreated with anti-miR-99a, and then adoptively transferred to naive recipients (n = 4 per group) that 24 hours later were challenged with OVA to elicit DTH reaction, measured as ear swelling 24 hours later. Data are expressed as delta ± SEM. One-way ANOVA with post hoc RIR Tukey test; ***P < 0.005, ****P < 0.001.
